# Taking real steps in virtual nature: a randomized blinded trial

**DOI:** 10.1007/s10055-022-00670-2

**Published:** 2022-07-06

**Authors:** Sigbjørn Litleskare, Fred Fröhlich, Ole Einar Flaten, Amelia Haile, Svein Åge Kjøs Johnsen, Giovanna Calogiuri

**Affiliations:** 1 Department of Public Health and Sport Sciences, Inland Norway University of Applied Sciences, Elverum, Norway; 2 Game School–Department of Game Development, Inland Norway University of Applied Sciences, Hamar, Norway; 3 Department of Psychology, Inland Norway University of Applied Sciences, Lillehammer, Norway; 4 Department of Nursing and Health Sciences, Centre for Health and Technology, University of South-Eastern Norway, Drammen, Norway

**Keywords:** Green exercise, Virtual reality, Immersive virtual environments, Virtual green exercise, Mixed-methods

## Abstract

Studies show that green exercise (i.e., physical activity in the presence of nature) can provide the synergistic psychophysiological benefits of both physical exercise and nature exposure. The present study aimed to investigate the extent to which virtual green exercise may extend these benefits to people that are unable to engage in active visits to natural environments, as well as to promote enhanced exercise behavior. After watching a video validated to elicit sadness, participants either performed a treadmill walk while exposed to one of two virtual conditions, which were created using different techniques (360° video or 3D model), or walked on a treadmill while facing a blank wall (control). Quantitative and qualitative data were collected in relation to three overarching themes: “Experience,” “Physical engagement” and “Psychophysiological recovery.” Compared to control, greater enjoyment was found in the 3D model, while lower walking speed was found in the 360° video. No significant differences among conditions were found with respect to heart rate, perceived exertion, or changes in blood pressure and affect. The analysis of qualitative data provided further understanding on the participants’ perceptions and experiences. These findings indicate that 3D model-based virtual green exercise can provide some additional benefits compared to indoor exercise, while 360° video-based virtual green exercise may result in lower physical engagement.

## Introduction

### The salutogenic effects of nature and green exercise

Research has shown that exposure to natural environments is important for human health and well-being. A recent study estimated that individuals who spend at least 120 min in contact with nature during a regular week achieve significant improvements of health and well-being (White et al. 2019). In this regard, green exercise (any physical activity in presence of nature; Pretty et al. 2003) is considered particularly beneficial, as one may combine the benefits of nature exposure with the benefits of physical activity. Compared to physical activity taking place indoors or urban settings, green exercise can provide more positive effects on indicators of exercise experience, physical engagement and psychophysiological recovery, for example: higher levels of enjoyment (Focht 2009), reduced sense of perceived exertion (Calogiuri et al. 2015; Harte and Eifert 1995), increased exercise intensity (Mieras et al. 2014), reduced blood pressure (Pretty et al. 2005), cognitive restoration (Kajosaari and Pasanen 2021), and improved mood or affect state (Calogiuri et al. 2015; Focht 2009; Hartig et al. 2003; Lacharité-Lemieux et al. 2015). There is still some debate regarding the underlaying mechanisms for the additional benefits of green exercise, but the recent theoretical framework by Rogerson et al. (2019) suggest that the psychological benefits associated with nature exposure (e.g., reduced mental-fatigue) interact with the physiological effects of exercise (e.g., reduced cortisol levels), eliciting additional health benefits compared to when these two stimuli occur independently (Rogerson et al. 2019). According to this framework, such psychological and physiological benefits support health by intertwining with behavioral factors, so that the resulting psychophysiological benefits shape exercise behavior, for example leading to higher exercise intensity (Mieras et al. 2014) and encouraging long-term exercise participation (Calogiuri and Chroni 2014). Unfortunately, because of a variety of reasons, including poor accessibility to safe and physical activity-supportive natural environments, challenging weather conditions, and individual barriers such as poor health, many people may not have the possibility to regularly engage in green exercise. For instance, a English study found that only about 20% of the population engaged in green exercise sessions of at least 30 min at least once during a regular week (White et al. 2016), while a Norwegian study found that about half of the population engaged in green exercise for at least one hour during a regular week (Calogiuri et al. 2016).

### Virtual green exercise

Virtual green exercise is defined as physical activity while being exposed to virtual representations of nature (Litleskare et al. 2020). Virtual green exercise aims to extend the benefits of nature exposure and green exercise to people that are unable to engage in active visits to real nature, such as residents in health care facilities or people with limited accessibility to natural environments, as well as to promote health through enhanced physical activity and nature exposure (Litleskare et al. 2020). For this reason, virtual green exercise received increased attention within healthcare (White et al. 2018). A recent integrative analysis by Calogiuri et al. (2021) proposed that virtual green exercise may provide health benefits in line with the framework proposed by Rogerson et al. (2019). In particular, by adding the element of nature exposure, virtual green exercise could deliver psychophysiological (e.g., cognitive restoration, mood enhancement and stress relief) and behavioral (e.g., enhanced exercise output) benefits above and beyond indoor exercise. The integrative analysis by Calogiuri et al. (2021), however, highlighted that, to date, the evidence regarding health benefits of virtual green exercise is still in its infancy. Accordingly, a recent systematic analysis by Lahart et al. (2019) found no evidence of consistent psychological or physiological benefits of virtual green exercise compared to indoor exercise. This review, however, emphasized the fact that studies in this field was associated with a high risk of bias, and called for more rigorous experimental trials on this topic.

### The issue of immersion and cybersickness

In the context of virtual green exercise, different types of technology have been used to create virtual representations of natural environments, including computer screens (see, e.g., Akers et al. 2012; Mayer et al. 2009; Pretty et al. 2005; White et al. 2015) and head-mounted displays ( HMD; see, e.g., Alkahtani et al. 2019; Calogiuri et al. 2018; Chan et al. 2021). By enclosing the user within the virtual environment and replacing the sensory input from the real surroundings with sensory input from the virtual environment (Slater et al. 1995), HMDs are believed to be a more efficient tool compared to other displays, as they can provide higher levels of immersion (Joseph et al. 2020; Litleskare et al. 2020). Immersion is defined as a technology’s capability of delivering an inclusive, extensive, surrounding, and vivid illusion of reality to the senses of a human participant (Slater and Wilbur 1997). Higher levels of immersion are believed to improve the VR experience as it contributes to increased levels of presence, i.e., the subjective feeling of being within the virtual environment (Slater and Wilbur 1997), ideally to such a degree that the virtual environment feels more real than the actual surroundings. Presence is considered a key element for the success of a virtual natural environment (Litleskare et al. 2020). In support of these assumptions, studies show that exposures to virtual representations of nature through HMDs can provides greater relaxation and stress relief compared to non-immersive technologies (Knaust et al. 2021; Liszio et al. 2018).

Unfortunately, highly immersive displays are also more prone to cybersickness (Chang et al. 2020; Guna et al. 2019; LaViola 2000; Sharples et al. 2008; Yildirim 2020). Cybersickness is considered a specific type of motion sickness and is known to cause symptoms such as dizziness, nausea and general discomfort (Kennedy et al. 2010; Smith 2015). The etiology of cybersickness is still unclear, but the two most prominent theories suggest that it is caused by either sensory conflict (Oman 1990; Reason 1978) or postural instability (Riccio and Stoffregen 1991) during VR immersion. Cybersickness may have a severe negative impact on experiences in VR and hamper the users’ feelings of presence (Weech et al. 2019) as well as distort research findings (Calogiuri et al. 2018). Recent research provides some insight into how to alleviate the issue of cybersickness. Synchronizing the treadmill speed with the optical flow (Chang et al. 2020; Saredakis et al. 2020) and minimizing scene oscillations (Litleskare and Calogiuri 2019) has been shown to reduce the severity of cybersickness.

### Challenges associated with virtual green exercise

The use of HMDs in studies of green exercise is considered a challenging endeavor, due to increased risk of inducing cybersickness in dynamic VR content, as well as the challenge of maintaining upright balance during virtual exercise (Joseph et al. 2020; Litleskare et al. 2020). To the best of the authors’ knowledge, only three published studies have been conducted on the effects of a dynamic virtual green exercise simulation using a HMD with a full field of view (Alkahtani et al. 2019; Calogiuri et al. 2018; Chan et al. 2021). Calogiuri et al. (2018) compared a real green exercise bout with a virtual homologue while simultaneously walking on a manually driven treadmill. While participants rated the two environments with equal levels of environmental restorativeness and spontaneously walked at the same pace in both conditions, the experience was severely influenced by cybersickness, which was associated with a decrement of participants’ affect and an increased sense of effort compared to the real green exercise bout (Calogiuri et al. 2018). Alkahtani et al. (2019) compared two high-intensity interval cycling sessions, with and without exposure to a video montage of natural scenes. The study found no significant differences between the two sessions for mood, thought there was some indication of greater psychological distress in the virtual green exercise condition (Alkahtani et al. 2019), possibly related to cybersickness. Chan et al. (2021) examined changes in mood, nature connectedness, and heart-rate variability in both young adults and elderly exposed to a virtual natural environment or a virtual urban environment, which they could navigate using a “walk in place” system. In this case, this study found that, compared with the virtual urban walk, virtual green exercise provided greater psychophysiological benefits.

### The impact of developmental techniques on virtual green exercise experiences

While the existing literature provides mixed findings regarding the possibility to use HMDs for virtual green exercise purposes, these mixed findings also suggest that the characteristics of hardware, software, mode of locomotion etc. are important to overcome the issue of cybersickness and enhance the overall user’s experience. In particular, the technique used to develop the VR scenery is emerging as an important factor influencing the experience. Mostly, two types of VR scenarios are uses for simulation nature experiences; 360° videos created using 360° cameras, or computer-generated virtual environments developed through three-dimensional (3D) modelling. The VR sceneries used in Calogiuri et al. (2018) and Alkahtani et al. (2019) consisted of 360° videos. As this technique reproduces photographic representations of actual environments, it can allow the creation of highly realistic virtual nature sceneries. On the other hand, a recent review have highlighted challenges associated with VR scenaries created using this technique, especially in relation to cybersickness (Saredakis et al. 2020). In recent years, 3D models based on video game development techniques have emerged as an alternative to 360° videos when creating representations of natural elements and landscapes. Advances within this field allow for creation of 3D models containing representations of nature that can achieve high levels of realism. Studies using this type of VR sceneries in the context of virtual green exercise have reported positive psychophysiological responses as demonstrated by Chan et al. (2021). However, to the best of the authors’ knowledge, only two studies have directly compared 360° videos with matching (or similar) 3D models. Nukarinen et al. (2020), compared physiological and psychological responses associated with exposure to a static 360° video of a forest versus a matching 3D model, with a third experimental condition in the real natural environment being also included. While no direct differences were found between the two VR conditions for any of the tested parameters, the study found a significant greater reduction in negative affect in the real nature condition as compared with the 360° video exposure, while such difference was not observed for the 3D model. Yeo et al. (2020) compared psychological responses associated with exposure to a static 360° video versus a 3D model, both showing a sequence of underwater tropical coral reef scenes. The findings of this study indicated equivalent effects of both VR sceneries for all psychological parameters included except for positive affect, which showed greater improvements in the 3D model as compared with the 360° video. In conclusion, although some evidence suggests that a sedentary exposure to a 3D model may elicit more positive affective responses than those elicited by 360° videos (Nukarinen et al. 2020; Yeo et al. 2020), such evidence is limited.

### The present study

The purpose of the present study was two-fold. Firstly, the study examined the participants’ perceptions and experiences associated with two virtual green exercise experiences created using different techniques (i.e., 360° video and 3D modeling) as compared to treadmill walking without VR exposure. Secondly, the study investigated the extent to which the two virtual green exercise conditions could elicit greater physical engagement and psychophysiological recovery compared to treadmill walking without VR exposure. By collecting and analyzing quantitative and qualitative information, the study was set to provide both statistical evidence in the context of a blinded experiment and in-depth understanding of the participants perceptions and experience.

## Materials and methods

This paper is structured in line with the CONSORT guidelines and the trial was pre-registered at ISRCTN (trial ID: ISRCTN14275608).

### Trial design

**Fig. 1:**
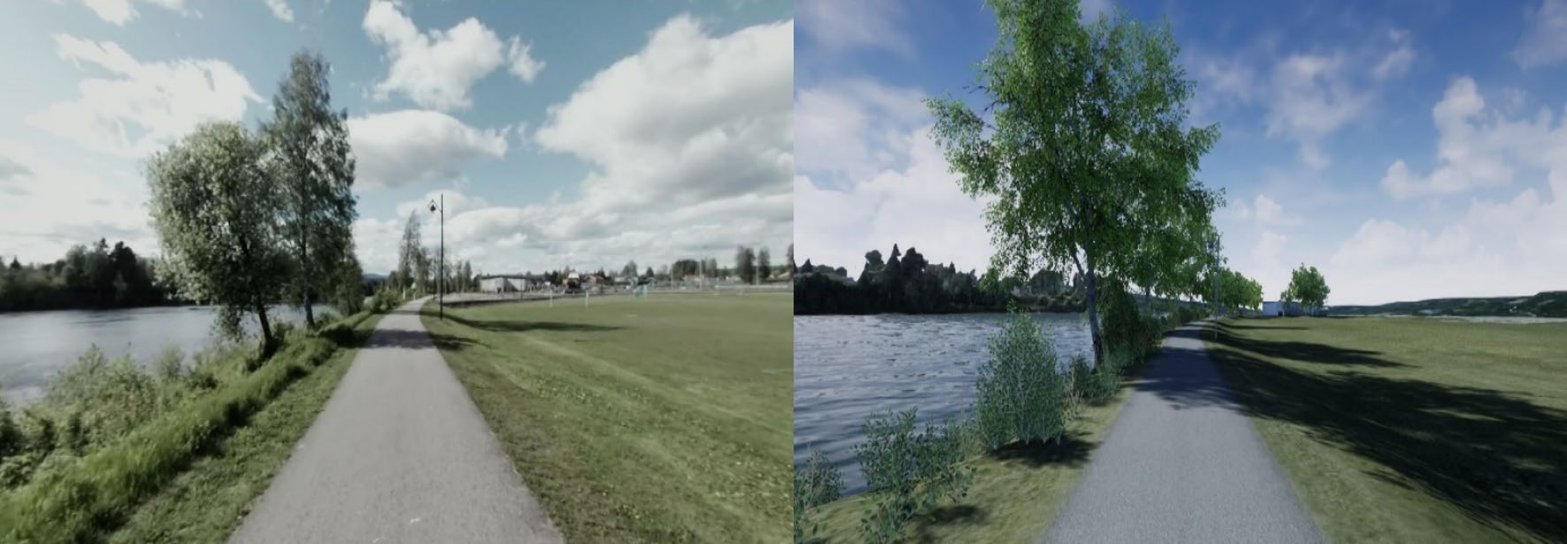
Snapshot from the 360° video (left) and the 3D model (right)

The study was designed as a double-blinded experimental trial with three parallel groups (Fig. 3) to reduce the impact of carry-over- and expectancy effects (Litleskare et al. 2020). The parallel group design is generally the recommended design for this type of VR study (Joseph et al. 2020; Litleskare et al. 2020). Participants were randomly assigned (picked from a hat) to one of the three experimental conditions. To minimize the expectancy effect in both the participants and the examiner, the randomization was performed after the baseline- and pre-exposure assessments (Fig. 1), meaning the participants and the examiner were blinded to the allocated condition during assessments at baseline and pre exposure, but not post exposure. The experiment was performed at a Sport Physiology Laboratory with standardized temperature (18 °C), ventilation, and lighting, and a high degree of sound insulation. No changes to methods were made after trial commencement.

### Participants

Estimation of required sample size was performed using the G*Power software, based on expected effect size for negative affect, which previous research has found to be a sensitive measure for detecting impacts of virtual nature (Frost et al. 2022; Yeo et al. 2020). The expected effect size was set as medium (f = 0.25), as previous research suggests that the typical effect of real green exercise on negative affect is equivalent of a medium effect size or larger (Lahart et al. 2019). Alpha was set to 0.05 and power was set to 90%. The optimal sample size was estimated to be 54 participants. Six additional participants were recruited to account for possible missing data. Participants (n = 60) were recruited via announcements on Facebook, the university’s official webpage, and by word of mouth. All participants were required to be 18 years or older, have normal or corrected to normal sight and without any diagnose of balance impairments. The study was approved by the Regional Ethics Committee for Medical and Health Research (ref. number 134663) and was performed according to the Declaration of Helsinki. The participants were informed about the purpose of the study and associated benefits and risks, before they gave their written consent to participate.

### Experimental conditions and VR technology

Three conditions were tested: (i) 360° video exposure; (ii) 3D model exposure; and (iii) control condition (walking on treadmill while facing blank wall). The two VR conditions (360° video and 3D model) reproduced the same environment, i.e., a walk by a river (Fig. 1). Previous research has shown that an actual walk along this path, in the same season and similar weather conditions, elicited positive emotional responses (Calogiuri et al. 2018). Participants viewed a 2′50’’ film clip that is validated to elicit the feeling of sadness prior to exposure to one of the three experimental conditions (Rottenberg et al. 2007). The purpose of this film clip was to reduce individual variation in affect prior to the experimental conditions and to invoke a state were the restorative potential of virtual green exercise may transpire. In all conditions, the participants walked on a manually driven treadmill (Woodway curve, Woodway Inc., USA) for 10 min at self-selected speeds (the participants were instructed to walk at a comfortable pace and to hold on to the hand-rails of the treadmill to maintain balance) while wearing the HMD or facing the blank wall. The manual treadmill allowed participants to walk at a self-selected speed to increase ecological validity (Focht 2009), and to assess their behavioral response to the different experimental conditions and the corresponding psychophysiological effects. The exposure duration of 10 min was chosen to comply with the minimum time to elicit positive psychological outcomes of real green exercise (Meredith et al. 2020). The walking speed in both VR-conditions was synchronized with the speed of the manually driven treadmill by using a USB output from the treadmill to obtain the necessary data. Synchronizing the treadmill speed with the optical flow of the playback should reduce cybersickness (Chang et al. 2020; Saredakis et al. 2020). The playback was made via a HTC Vive Pro HMD (resolution of 2880 × 1600; refresh rate of 90 Hz) connected to a computer (Intel(R) i7-8700 k processor, 16 gigabytes of RAM, NVIDIA Geforce RTX 2080 graphics card), and Sony WH-1000X M3 noise canceling headphones (Sony Corporation, Tokyo, Japan). There was no VR control condition, as research shows that there is no difference between virtual control conditions and real control conditions (Yin et al. 2018).

The 360° video was developed using a GoPro Fusion 360° camera with a built-in stabilizer (5228 × 2624, resolution, 30 frames per second; GoPro, San Mateo, California, USA). Following the approach by Litleskare and Calogiuri (2019), the camera operator was moving along the path on a hover board (AAG, MADD gear electric, Victoria, USA) to improve the stability of the recording which should reduce cybersickness severity (Litleskare and Calogiuri 2019). The video was then run through a post-production editing process. First, the video was edited in the GoPro Fusion Studio (GoPro Inc., California, USA) to apply the Full Stabilization filter, which locks the orientation of the camera on the horizon. The edited video was imported to Adobe Premiere Pro (Adobe Systems, California, USA) to adjust the colors for a more realistic look compared to the slightly over-saturated raw video clip. The 3D model was created using photogrammetry techniques and assembled for real time playback in Unreal Engine 4.22 (Epic Games, Cary, North Carolina, USA). A high-resolution digital terrain model obtained from hoydedata.no served as the basis to accurately recreate the general landscape, elevation, and horizon. The path and immediate surroundings were scanned with a drone in 4 K resolution (Phantom 4 Pro UAV, DJI, Shenzhen, China). The 3D model was reconstructed from the aerial drone photographs with the photogrammetry software RealityCapture (Capturing Reality, Bratislava, Slovakia). The photogrammetry data was optimized for real time playback, and the retopologizing and UV mapping was done using the software 3D Coat version 4.8 (Pilgway, Kiev, Ukraine). Other objects like lamps and trash bins were reconstructed with photo references using the digital content creation application Maya (Autodesk, San Rafael, California, USA). Bushes and grass models are based on purchased presets from SpeedTree (Interactive Data Visualization Inc., Lexington, South Carolina, USA). The optimized 3D assets were imported into Unreal Engine where a lighting model was created to match the geolocation. Due to performance issues, some minor elements from the real location were not recreated in the 3D model (e.g., grass/foliage by the river, see Fig. 1). A surround microphone that captures sound in four channels simultaneously was used to record sounds along the path (Zoom H2, Zoom Corporation, Chiyoda-ku, Japan). This soundtrack was used for both the 360° video and the 3D model played back in full surround sound based on spatial audio and the users head movement.

To adhere to current recommendations regarding descriptions of the specific elements present in the environment (Browning et al. 2020a, b), two videos of our prototype environments were uploaded to YouTube (https://www.youtube.com/watch?v=hK3vzKaHDao and https://www.youtube.com/watch?v=8VKzMnU9Tno). Although the content of the environments is representative of the final version, the experimental setting, procedures, and conditions are not.

### Instruments

#### Experience

*Enjoyment* – The participants level of enjoyment was assessed after the experimental condition using the following inquiry: “on a scale from 1 to 10, how enjoyable was the activity you engaged in?” This measure has previously been used in studies examining affective responses to green exercise (Calogiuri et al. 2015, 2018).

*Perceived environmental restorativeness –* The perceived restorativeness scale (PRS; Hartig et al. 1997; 2003) was used as a measure of (virtual) environmental qualities that may lead to psychological restoration. This scale consists of 16 items assessing the subjective perception of four environmental qualities in line with the Attention-restoration theory (R. Kaplan 1989; S. Kaplan 1995): fascination, the environments ability to capture the viewers’ effortless attention (five items, e.g., “The setting has fascinating qualities”); being away, the environments ability to provide a feeling of “being away” from everyday demands and concerns (two items, e.g., “It was an escape experience”); coherence, the extent to which the elements in an environment is perceived as a coherent whole (four items, e.g., “There is too much going on”); and, compatibility, the extent to which the environment is compatible with the participants inclinations and interests (five items, e.g., “have a sense that I belong there”). The internal consistency for the different components were adequate for fascination (α = 0.87), coherence (α = 0.78) and compatibility (α = 0.78), but somewhat poor for being away (α = 0.66).

*Cybersickness –* The severity of cybersickness symptoms were assessed by the simulator sickness questionnaire (Kennedy et al. 1993) after the three conditions. The questionnaire was originally developed to measure simulator sickness, but has been extensively used in studies of cybersickness as well (Chang et al. 2020). Participants were asked to rank the severity of 16 different symptoms on a 4-point Likert scale. The total score of these symptoms was calculated according to the recommendations of Kennedy et al. (Kennedy et al. 1993). The scale showed adequate internal consistency for the total SSQ score (α = 0.75).

*Presence –* The assessment of the participants’ sense of presence in the two virtual environments was based on the approach of Nichols et al. (Nichols et al. 2000), but slightly modified to fit the purpose of this study. The participants were asked to rate the level of agreement to eight statements related to the presence in virtual environments, using a 11-point Likert scale, as previously used in Litleskare and Calogiuri (Litleskare and Calogiuri 2019).

### Physical engagement

*Walking speed –* The walking speed was recorded during the full 10 min of the experimental conditions by the built-in treadmill computer. The average walking speed used for further analysis.

*Heart rate –* Heart rate (HR) was recorded continuously during the treadmill walk via a HR-monitor (Garmin Forerunner 310XT, Garmin International Inc., Olathe, Kansas, USA) and extracted as beats per minute. The mean of all individual measurements was automatically recorded by the HR-monitor and used for further analyses.

*Perceived exertion –* Participants reported their ratings of perceived exertion (RPE) immediately after completing the experimental condition using the 20-point version of the Borg scale with verbal cues (Borg 1982). The scale consists of values ranging from 6–20 with a corresponding description of the level of exertion (e.g., 11 = fairly light).

### Psychophysiological recovery

*Affect –* Participants’ affect was assessed at baseline (i.e., before the stress-elicitation), pre-, and post-exposure to the experimental conditions using the physical activity affect scale (PAAS; Lox et al. 2000). The scale consists of 12 items (e.g., “energetic”, “calm”, “miserable”, and “tired”) that are grouped in four components according to Russel’s circumplex model of affect (Russell 1980): positive affect (positive valence, high activation), tranquility (positive valence, low activation), negative affect (negative valence, high activation), and fatigue (negative valence, low activation). The scale showed adequate internal consistency for most of the components at most time points (α = 0.65–0.80); however, poor levels of internal consistency were found for positive affect at baseline (α = 0.39) and pre (α = 0.57).

*Blood pressure –* The Watch BP Office Target semi-automatic blood pressure kit (Microlife, Taipei, Taiwan) was used to measure blood pressure at four different time points; after five minutes of sedentary time at baseline; at 5 min after viewing the film clip designed to elicit sadness; at 5 and 15 min after completing the experimental condition, as a previous study has shown that blood pressure was significantly lower 15 min after exercise in a projection-based virtual green exercise trial (Duncan et al. 2014).

### Participants’ background characteristics

Information regarding the participants’ sex, age, body mass index (BMI), physical activity habits, and connectedness to nature was collected and used as background characteristics to describe the participant’s health status, levels of physical activity, and inclinations toward nature. Sex, age, and height were self-reported by the participants (due to Covid-19 restrictions), while weight was measured using a Seca 877 (SECA GmbH, Hamburg, Germany). The participant’s physical activity levels were assessed using a version of the leisure time exercise questionnaire (LTEQ; Godin and Shephard 1985) modified to include transportation physical activity (i.e., walking or biking as a means of transportation). This adjusted version of LTEQ correlates well with assessments of physical activity by accelerometer (Calogiuri et al. 2013). The participants baseline level of nature connectedness was assessed by a version of the connectedness to nature scale (CNS) modified to measure state (Mayer et al. 2009).

### Qualitative information

As little is known about how people perceive and respond to virtual green exercise, in addition to the quantitative data described above, qualitative information was collected among the participants who underwent one of the two VR conditions. This allowed to capture possible themes not dominated by a priori categories, as well as to gain a more nuanced understanding of the quantitative findings. The qualitative information was collected in form of written essays guided by open-ended questions, which were presented to the participants after completing the VR conditions and subsequent quantitative assessments. In order to facilitate the integration of the quantitative and qualitative findings, the questions were developed based on the overarching themes of the quantitative assessments- a question was developed for each of the quantitative variables in such way that the participants could elaborate further on the responses provided in the questionnaire. Questions were phrased to allow for negative, positive as well as neutral viewpoints, for example: “What emotions are you experiencing right now, after having completed the virtual walk?” and “When you answered the question about how “enjoyable” the activity was, what determined where on the scale you put your mark?”.

### Procedures

**Fig. 2:**
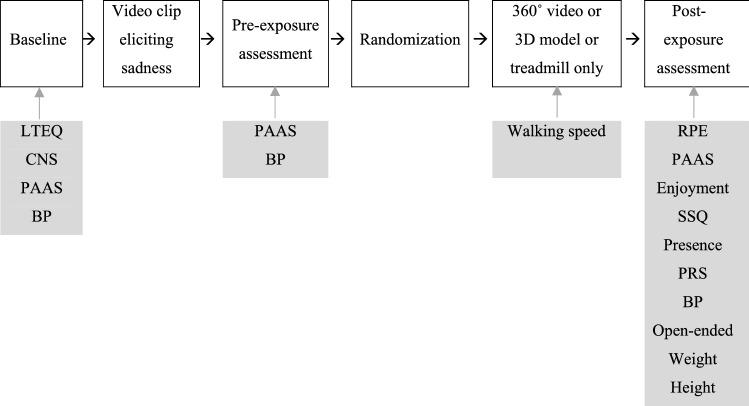
Overview of experimental procedures (LTEQ = leisure time exercise questionnaire; CNS = Connectedness to nature scale; PAAS = physical activity affect scale; BP = blood pressure; RPE = rating of perceived exertion; SSQ = simulator sickness questionnaire; PRS = perceived restorativeness scale)

An overview of the experimental procedures is presented in Fig. 2. After meeting at the laboratory, the participants remained seated for 5 min while completing the baseline questionnaire, which included the LTEQ, CNS, and PAAS. After completing the questionnaire, a baseline measurement of blood pressure was taken and the participants subsequently watched the film clip validated to elicit feelings of sadness (Rottenberg et al. 2007). The participants then remained seated for 5 min while completing the pre-exposure PAAS assessment, after which the pre-exposure measurement of blood pressure was taken. Next, the participants were randomly allocated to one of the three conditions, by picking a number from a hat, and the participant received the information corresponding to that condition only. Participants were briefly familiarized with the treadmill and the HMD before performing the allocated condition. After completion of the treadmill walk, the post-exposure questionnaire was administered, which consisted of RPE, PAAS, enjoyment, SSQ, presence, PRS, and the open ended questions (the three latter were administered only to the participants in the two VR-conditions). Blood pressure was measured 5 and 15 min after completion of the treadmill walk. Lastly, the participants were weighed and reported their height to the closest centimeter. All measurements were performed according to standard procedures for each instrument. The questionnaires included additional questions regarding future green exercise intention and nature connectedness that are not presented in this paper.

### Analyses

To analyze the quantitative findings, one-way ANOVA with post hoc analysis was used to assess possible effects of “condition” (360° video, 3D model and control) on enjoyment, SSQ, and walking speed. An ANCOVA with post hoc analysis was used to analyze possible effects of “condition” on HR and RPE, while correcting for walking speed. An independent-samples Student’s t-test was used to analyze potential differences between the two VR conditions for PER and presence. A mixed between-within subjects ANOVA with post hoc analysis was used to test for possible effects of “time” (baseline, pre exposure, post exposure) and “time by condition” interaction on the different components of PAAS and blood pressure. All post hoc analyses were performed applying a Bonferroni’s correction of alpha. The statistical approach was in accordance with the strategy set prior to experiments. The results are presented as means and standard deviation (M ± SD). The level of significance was set at *p* < 0.05. The statistical analyses were performed in SPSS version 25 (IBM corp., New York, USA).

The purpose of the qualitative analysis to extend and deepen the understanding of the quantitative findings. The qualitative data were analyzed thematically, with an coding frame being defined a priori based on the main domains of the quantitative instrument (i.e., experience, physical engagement, and psychological recovery) and the psychological constructs contained within each of these overarching themes (e.g., enjoyment, perceived exertion, and changes in psychological states). Reiterative reading and recoding of the data led to refinement and extension of the coding frame. In this way, the themes that emerged from the analysis were substantively grounded in the data, while being informed by the domains of the quantitative instrument, which served the purpose of facilitating the following integration between quantitative and qualitative findings. To be noted that, although the qualitative analysis was *informed* by the quantitative constructs, the emerged themes are independent of the quantitative findings. A first draft of the analysis was performed by one author (GC), which was then further developed in collaboration with another author (SÅKJ). A third author (AH) acted as a “critical friend.”

In the Results chapter, for each overarching theme (experience, physical engagement, and psychological recovery), the quantitative and qualitative findings are presented separately and independently from each other—i.e., first the outcomes of the statistical analysis are presented, followed by the themes emerged from the qualitative analysis, without any integration between the two strands. The integrated discussion of the quantitative and qualitative findings is eventually presented in the Discussion chapter, with emphasis on the similarities and differences of these two approaches. The quantitative findings shall be seen as having most weight, especially with respect to providing statistical evidence of the effectiveness of the VR conditions as compared with treadmill walking alone as well as possible differences between the two types of VR (360° video vs. 3D model). Although the qualitative findings shall be seen as secondary, they are important to enhance and extend the understanding of the quantitative findings, especially with respect to the participants’ perceptions and experiences.

## Results

**Fig. 3:**
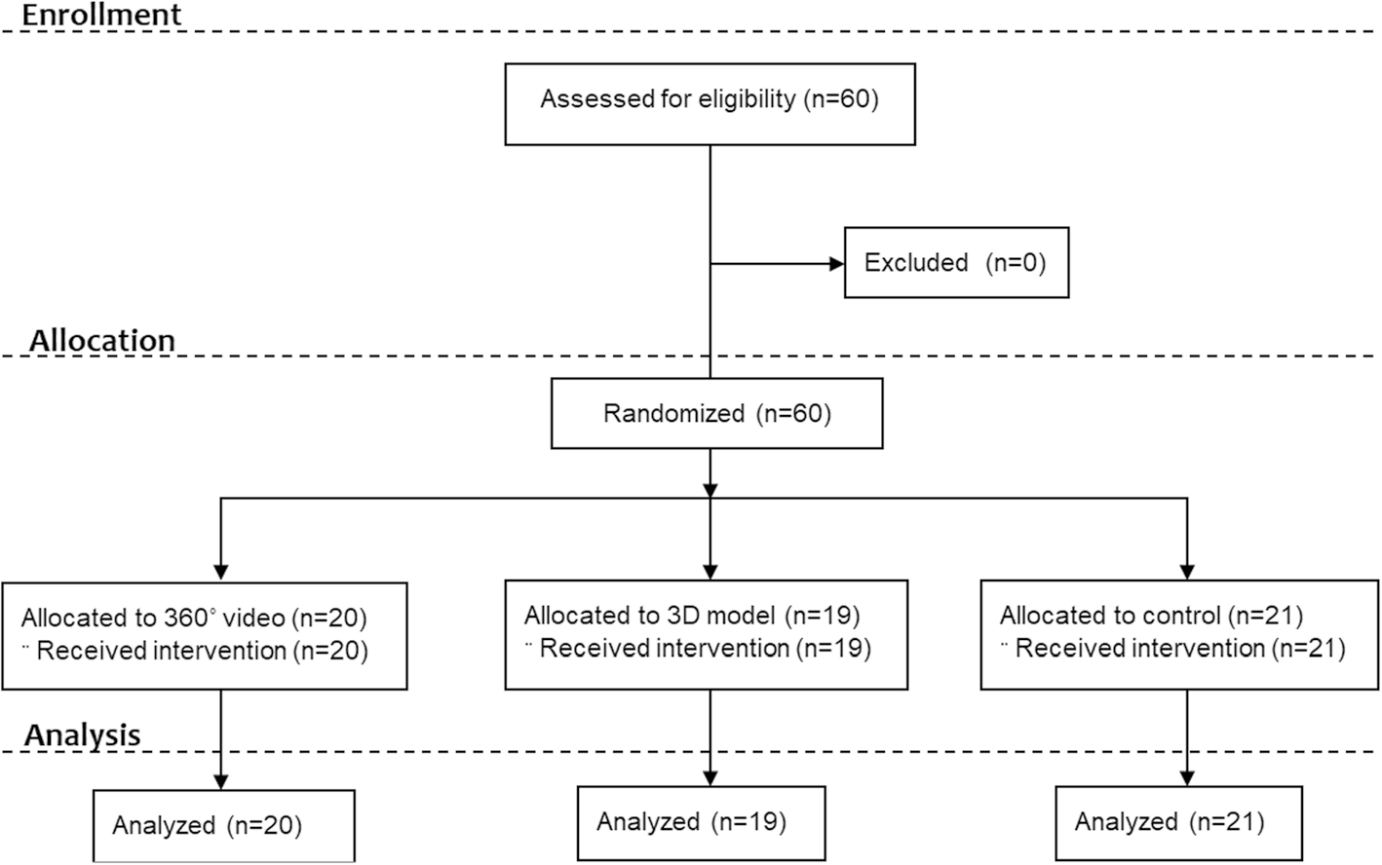
Flow diagram showing the number of participants enrolled, allocated to experimental conditions and included in the analysis

Study enrolment and allocation are presented in Fig. 3. The participants in this study were considered healthy and active individuals with fairly high levels of connectedness to nature (Table 1).

**Table 1:** Gender distribution, age, body mass index (BMI), levels of physical activity (LTEQ—leisure time exercise questionnaire) and levels of nature connectedness (CNS—connectedness to nature scale) for all participants in each experimental condition

	360˚ video (*n* = 20)	3D model (*n* = 19)	Control (n= 21)
Males/females (*n*)	12/8	9/10	11/10
Age (years)	31.2 ± 13.7	31.6 ± 15.3	27.1 ± 7.3
BMI (kg/m^2^)	24.8 ± 3.8	24.8 ± 4.0	25.0 ± 2.6
LTEQ	58.6 ± 22.9	54.9 ± 24.9	57.1 ± 28.3
CNS	3.9 ± 0.7	4.0 ± 0.7	4.0 ± 0.6

Means ± standard deviation

### Experience

**Fig. 4:**
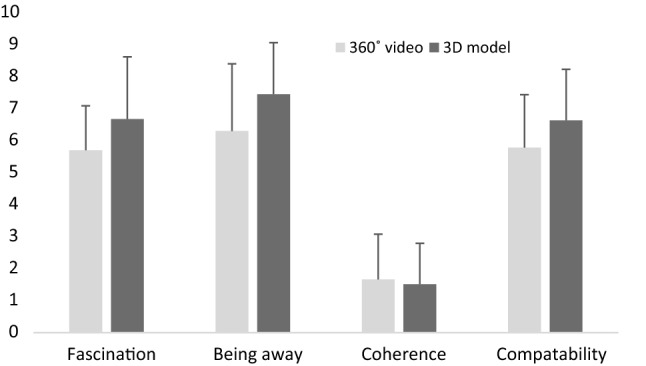
Ratings of the four components of the perceived restorativeness scale in the 360° video and the 3D model (M ± SD). NB: Low values of “coherence” indicate high perceived coherence

**Fig. 5:**
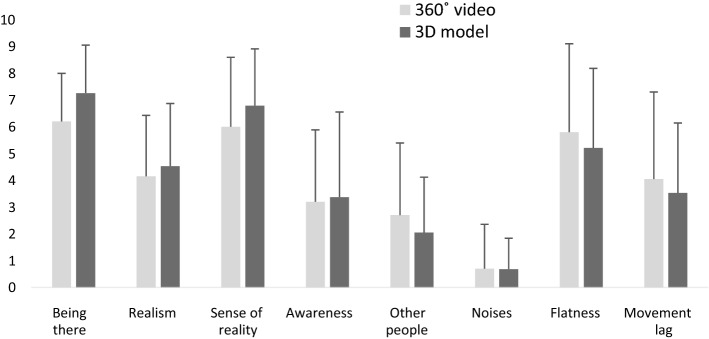
Ratings of presence in the 360° video and the 3D model (M ± SD)

The mean values of enjoyment were 6.9 ± 2.4 in the 360° video, 8.3 ± 1.9 in the 3D model, and 5.9 ± 2.6 in control. The one-way ANOVA revealed a significant effect of condition on enjoyment (F(2,57) = 5.357, *p* = 0.007, ηp = 0.158), with post hoc analysis demonstrating that the 3D model was perceived as more enjoyable compared to control (*p* = 0.006). No significant differences were found between 360° video and control (*p* = 0.533) or between 360° video and 3D model (*p* = 0.189). Overall, satisfactory ratings were observed for all the PRS components (Fig. 4). No significant differences between the two VR conditions were found for any of the PRS components (fascination: t(37) = -−81, *p* = 0.078; being away: t(37) = -−91, *p* = 0.064; coherence: t(37) = 0.34, *p*  = 0.733; compatibility: t(37) =  −1.64, *p* = 0.110). The SSQ score was 25.2 ± 15.6 in the 360° video, 21.1 ± 15.0 in the 3D model, and 16.2 ± 14.7 in control, indicating relatively low levels of cybersickness. The ANOVA showed no significant differences among conditions in relation to SSQ score (F(2,57) = 1.801, *p* = 0.174, ηp = 0.059). Rather high levels of presence, especially in relation to “being there” and “sense of reality”, were found in both VR conditions, though rather high ratings for “flatness” and “movement lag” were also reported (Fig. 5). The t-test showed no significant differences between the 360° video and the 3D model for any of the eight items of presence (*p* > 0.05 for all eight items), but there was a tendency for the item “being there” in favor of the 3d model (t(37) = -−1.85, *p* = 0.072).

In relation to the overarching theme “2.4”, for each of the quantitative domains that initially informed the analysis, two sub-themes emerged: “excitement versus. boredom” and “break the routine” (enjoyment); “appreciating the naturalistic scenario” and “familiarity with the place” (environmental qualities); “dizziness negatively affecting the experience” and “challenges with postural stability” (cybersickness); “feeling Immersed” and “not like real nature” (presence).

It appeared that most of the participants enjoyed the experience, which was recurrently labelled as “fun,” “exciting”, or “interesting”. However, in both VR conditions, a few participants described the virtual walk as little stimulating (“A little boring”, “There was little going on”), while some participants appeared to be more excited by the novelty of the technology. This was especially the case among participants who never had tried VR before (e.g., “It was fun to try something new”; “The technology was fascinating”), but also by one who had previous familiarity with VR:

“VR is something I have tried before, and being able to actually move over longer distances while it happens in VR was a new and exciting experience… the simulation itself was not very impressive, but the movement aspect made the VR experience new and exciting.” (Man, 32 years, 360° video).

Some participants stated that the virtual walk gave them an opportunity to “disconnect” from daily routines in such a manner that was remarkably similar to what is associated with experiences in real nature. A few participants (all in the 3D model condition) described experiences that are indicative of a flow-like state:

“For a few minutes I almost forgot where I was and just focused on walking and did not think about anything else.” (Woman, 36 years, 3D model).

Although some participants found the virtual environment rather plain and little stimulating, several participants made explicit references to the fact that the environment (and most recurrently, the *nature* elements) triggered their curiosity, as exemplified by these quotations:

“There was little to look at. I felt like there was nothing I could focus on, as opposed to being in a forest where one can see new trees/animals/lakes around every corner” (Man, 22 years, 360° video).

“What caught my attention during the walk were the trees, the buildings, but also the river that flowed slowly past” (Woman, 24 years, 3D model).

The nature elements in the virtual environments were also recurrently associated with liking the setting (e.g., “The weather, the birds’ singing and the river’s rushing sound. Green grass and leaves [made the experience enjoyable for me]”), as opposed to elements of the built environment (e.g., “The less traffic noise the more pleasant”; “It was not very fascinating due to the large football pitches”). Several participants reported to have enjoyed the fact that the VR settings gave them an opportunity to experience a day of spring/summer and nice weather (“… it was nice to walk in daylight, now that it is dark early in the afternoon”).

Many participants also mentioned the fact that they were familiar with the place, which was associated with pleasant feelings of safety (“It was a safe and friendly environment”) and attachment with the place (“I felt a sense of belonging”).

“I ‘walked’ in a familiar environment where I often find myself and it was interesting to see it this way… I was in a place where I often visit and with which I have good relationships… The [virtual] environment attracted my attention because I have often visited that place myself, and quickly recognized myself there … I felt a sense of belonging because I often visit the virtual environment even in real life.” (Man, 22 years, 360° video).

Some participants (all in the 360° video condition) experienced dizziness during the VR experience. In most cases, the feelings of dizziness appeared relatively mild and/or temporary, though for two participants it was more severe:

“[I felt] a little uncomfortable… was a bit dazed and a bit dizzy during the walk and the last minutes I was just looking forward to be finished.” (Woman, 26 years, 360° video).

A few participants, in both the VR conditions, reported some challenges in relation to postural stability. It should be noted, however, that while the two participants in the 3D model condition appeared to have experienced relatively mild and/or temporary challenges, the experience of the one participant in the 360° video condition was more severe:

“I held on pretty tight with my hands [on the handrails] and certainly could not have managed to walk without holding myself … may have felt a little discomfort with my sight … during the [first half of the walk] it was easier to walk than the [last half].” (Woman, 20 years, 360° video).

About one third of the participants (and slightly more frequently in the 3D model condition), described experiences indicative of very high levels of presence, with several reporting that they felt disconnected from the real place (i.e., the laboratory room) and, in some cases, even appeared to be completely present in the virtual environment:

“I felt like I was out in the virtual environment and focused on it, so I did not know what was happening in the room… It was fascinating how real it was … so I felt like I was walking for real.” (Man, 22 years, 360° video).

The most frequently reported factors contributing to reduced feeling of being in nature were poor graphics, feelings of mismatch between one’s movements and those of their virtual selves’, and poor soundscape or presence of external noises (e.g., the noise from the treadmill). A few participants mentioned the lack of a virtual body, or that they experienced some discomfort in relation to the equipment (e.g., the weight of the headset). Several participants complained that the virtual world appeared visibly artificial (e.g., “It was so clear it was not real”), and some also noticed the lack of sensorial elements typically associated with experiences in real natural environments, such as feeling smells and the wind blowing (e.g., “It was … not something I would do again as I like to feel the smell and wind of nature when I walk”), but also the absence of other people:

“The only thing I might have missed a bit in the virtual environment was more activity from people and/or animals, as I felt quite alone… the virtual environment seemed a bit cold and lonely” (Man, 36 years, 3D model).

### Physical engagement

The mean walking speed was 6.6 ± 1.4 km/h in the 360° video, 7.7 ± 1.5 km/h in the 3D model, and 8.3 km/h ± 1.8 in control. The one-way ANOVA revealed an effect of condition on walking speed (F(2,57) = 6.694, *p* = 0.002, ηp = 0.190), with the post hoc analysis showing a significantly lower walking speed in the 360° video condition compared to control (*p* = 0.02), and a potential trend compared to the 3D model (*p* = 0.064). No statistically significant differences were found between the 3D model and control (*p* = 0.762). The ANCOVA found no differences among conditions for HR (98.4 ± 17.4 beats/min in the 360° video, 112.6 ± 16.6 beats/min in the 3D model and 121.8 beats/min ± 23.8 in control; (F(2,56) = 2.345, p = 0.110, ηp = 0.08) or RPE (9.9 ± 3.0 in the 360° video, 11.2 ± 2.2 in the 3D model and 10.5 ± 2.5 in control; F(2,56) = 1.284, *p* = 0.285, ηp = 0.04) when adjusting for walking speed. Three themes emerged with respect to the overarching theme “2.4.1”: “challenges with walking on treadmill,” “appreciating the possibility of self-pacing,” and “feeling the exercise.” Some participants, all in the 360° video condition, experienced some challenges with balance and walking on the treadmill, something that may have forced the participants to walk at a slower pace (e.g., “It was difficult to increase the speed”).

“I held on rather strongly with my arms [on the handrails] and certainly could not manage to walk without holding myself. I felt like I worked-out more with my arms than my legs in the end … the [first part] was easier to walk than the [second part]” (Woman, 20 years, 360° video).

On the other hand, the possibility of self-pacing appeared to be appreciated by other participants, who perceived it as something that allowed them to have control of the experience and enjoy the surroundings:

“I liked that one could walk at the pace one wanted, and it was a big plus. I’m used to VR experiences that just move at a set rhythm.” (Man, 29 years, 360° video).

“… as I could adjust my pace it was possible to look around” (Woman, 67 years, 3D model).

Some participants, all in the 3D model condition, reported to have experienced moderate levels of physical exertion:

“It feels a bit like I've been out for a little walk” (Woman, 29 years, 3D model).

“I feel in my body that I walked” (Man, 25 years, 3D model).

### Psychophysiological recovery

**Fig. 6:**
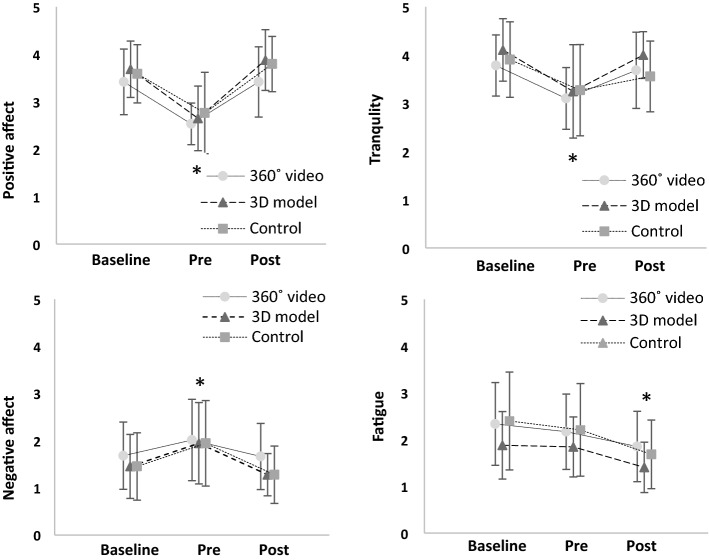
Ratings (M ± SD) of the four components of the physical activity affect scale in the 360° video, 3D model and control (means and standard deviations). Pre-values were significantly different from baseline and post for positive affect (top left), tranquility (top right) and negative affect (bottom left), while post-values were significantly different from baseline and pre for fatigue (bottom right)

**Fig. 7:**
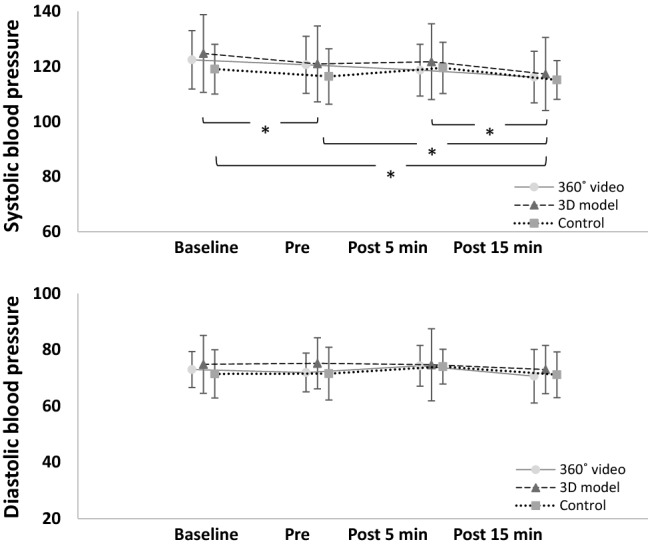
Values (M ± SD) for systolic (top) and diastolic (bottom) blood pressure at four different time points; baseline, pre, post 5 min and post 15 min. Systolic blood pressure was significantly reduced at post 15 min compared to baseline, pre and post 5 min, and systolic blood pressure at pre was significantly reduced compared to baseline. No significant differences were observed for diastolic blood pressure

Figure 6 shows the ratings (M ± SD) for all the components of affect in the different assessment points and different experimental conditions. The mixed between-within subjects ANOVA revealed a significant effect of “time” on all the components of affect: positive affect (F(2,114) = 56,71, *p* < 0.001, ηp = 0.499), tranquility (F(2,114) = 18,79, *p* < 0.001, ηp = 0.248), negative affect (F(2,114) = 15,69, *p* < 0.001, ηp = 0.216), and fatigue (F(2,114) = 18,45, *p* < 0.001, ηp = 0.245). However, no significant “time by condition” interaction was found for any of the components of affect (positive affect: F(2,56) = 2.376, *p* = 0.102, ηp = 0.018; tranquility: F(2,56) = 0,736, *p* = 0.483, ηp = 0.023; negative affect: F(2,56) = 1.399, *p* = 0.255, ηp = 0.017; fatigue F(2,56) = 2.178, *p* = 0.123, ηp = 0.015). A post hoc analysis showed that pre-exposure values were significantly lower compared to both baseline and post exposure for positive affect (baseline vs. pre: *p* < 0.001; pre vs. post: *p* < 0.001) and tranquility (baseline vs. pre: *p* < 0.001; pre vs. post: *p* < 0.001). A reversed pattern emerged for negative affect, as pre values were significantly higher compared to both baseline and post (baseline vs. pre: *p* = 0.001; pre vs. post: *p* < 0.001). The results for fatigue followed a slightly different pattern, with significantly lower values in post exposure compared with pre and baseline (post vs. baseline: *p* < 0.001; post vs. pre: *p* < 0.001), indicating a reduction in feelings of fatigue throughout the three assessment time-points. In regards to blood pressure (Fig. 7), the mixed between-within subjects ANOVA found a significant effect of “time” for systolic blood pressure (F(3,171) = 13,55, *p* < 0.001, ηp = 0.192), but not a significant “time by condition” interaction (F(6,171) = 1.21, *p* = 0.310, ηp = 0.041). The post hoc analysis showed a significant difference between baseline versus pre (*p* = 0.016), baseline versus. 15 min post exposure (*p* < 0.001), pre versus. 15 min post exposure (*p* = 0.004), and 5 min post exposure versus. 15 min post exposure (*p* < 0.001). No significant effects of “time” (F(3,171) = 2.11; *p* = 0.101, ηp = 0.036) or “time by condition” interaction were found for diastolic blood pressure (F(6,171) = 0.374, *p* = 0.895, ηp = 0.013).

From the qualitative analysis, in relation to the overarching theme “psychophysiological recovery,” two themes emerged: “enhanced positive affect,” and “relaxation.” While only a few participants, all in the 360° video condition and all men, reported to not have perceived a substantial change in their emotional state from before to after the virtual walk (“I feel pretty much like before doing the [virtual walk]”), many more, and in both conditions, made explicit references to experiencing positive emotional states as a result of the virtual walk. Such emotional states were identified as either within the domain of positive affect (high arousal and positive valence, e.g., “excited” or “energetic”) or within the domain of tranquility (low arousal and positive valence, e.g., “relaxed” or “calm”). The voice of one participant depicts the impact of the virtual walk in facilitating the psychological recovery after viewing the sad movie:

“[I feel] happy. Little surprised of how quickly I forgot about the sad movie” (Woman, 35 years, 3D model).

## Discussion

### Effectiveness of virtual green exercise

The overall results of this study show that, by using appropriate techniques to develop virtual natural environments, virtual green exercise can provide psychophysiological benefits with negligible side effects. In particular, the quantitative findings indicate that virtual green exercise was associated with high levels of enjoyment, relatively high levels of perceived environmental restorativeness and presence, and improvements in affect and blood pressure. The qualitative findings supported and extended these findings, indicating overall positive experiences, with participants reporting positive emotions (either in form of relaxation or positive affect), excitement, and feelings of “breaking from the routine.” Moreover, the qualitative reports emphasized how participants appreciated viewing naturalistic scenarios and/or familiar places, which triggered curiosity and sense of belonging. The qualitative findings also supported the quantitative findings regarding presence, indicating that participants felt like “being in” the virtual world, and in some few cases even completely loose the sense of reality (i.e., forgetting they were in the laboratory). Altogether, these findings indicate an improvement of the virtual conditions compared to those used in previous studies, which typically report adverse effects of virtual exposure (Alkahtani et al. 2019; Browning et al. 2020a, b; Calogiuri et al. 2018; Frost et al. 2022; Mostajeran et al. 2021). In the present study, both VR conditions were associated with low levels of cybersickness symptoms, as indicated by the similar SSQ ratings in the VR conditions as compared to control, but also by the fact that only few participants mentioned experiencing cybersickness (either in form of dizziness or impaired postural stability) in the qualitative reports. In this regard, it should be noted that some of the items of the SSQ, such as fatigue and sweating, can be influenced by the participants’ responses to physical exercise. Some participants walked at such speeds that they started sweating despite the conservative temperature in the laboratory (18˚C), and this may have inflated the reported SSQ scores. The relatively low prevalence and severity of cybersickness observed in the present study compared to previous studies (Calogiuri et al. 2018), may be attributed to high levels of scene stability and matching of walking speed between the treadmill and the movement in the virtual environments (Chang et al. 2020; Litleskare & Calogiuri 2019; Saredakis et al. 2020).

### Effects of virtual green exercise compared to control

The quantitative analysis revealed that virtual green exercise elicits higher levels of enjoyment compared to control (though, significant only in the 3D model condition). This finding is in line with previous studies on real green exercise versus. indoor exercise, which generally report higher levels of enjoyment after green exercise (Focht 2009; Lahart et al. 2019). Our results demonstrate that this effect may extend to virtual green exercise as well. The positive effects of virtual nature exposure on enjoyment was further bolstered by the large effect size (ηp = 0.158) and qualitative reports that highlighted how the natural elements in the virtual environments were recurrently associated with enjoyment and even flow-like states. Since enjoyment is a strong motive for exercise participation (Dishman et al. 1985), also in the specific context of green exercise (Calogiuri & Chroni 2014), these results support the idea that virtual green exercise might be a useful tool to increase exercise participation (Litleskare et al. 2020).

In line with previous research, the present study found limited evidence of other additional benefits of virtual green exercise compared to control (Lahart et al. 2019). Apart from enjoyment, no additional benefits of virtual green exercise were observed among the quantitative measurers, including perceived exertion, heart rate, and psychophysiological recovery in the form of pre-post changes in affect and blood pressure. This suggest that the beneficial changes in affect and blood pressure after the VR conditions were primarily associated with the physical activity rather than the virtual nature experiences. These findings challenges previous analysis proposing that synergic benefits occur when combining physical activity and (virtual) nature exposure, leading to greater benefits compared to when each of these occur individually (Calogiuri et al. 2021). In this regard, one important issue to consider is whether virtual nature can elicit restorative effects similar to real nature. Some researchers have found that nature experiences mediated through technology is not as restorative as real nature experiences (Kahn et al. 2008), while others show that virtual nature experiences can be restorative (Liszio et al. 2018). In the present study, exposure to both virtual environments elicited fairly high levels of perceived environmental restorativeness, which were contrasted by qualitative reports referring to the virtual environments as artificial. These reports were not only related to graphics, but also to lack of detail and lack of sensorial elements that are typically associated with real nature. This was recurrently associated with lower ratings of presence and, in some cases, a general feeling that the virtual environment could not reproduce an authentic experience of real nature. Thus, the virtual environments limited ability to fully reproduce an authentic nature experience may impede the associated psychophysiological outcomes compared to real nature.

### Differences between 360˚ video and 3D model

To date, very little published evidence exists on the way users perceive and respond to virtual green exercise settings developed with different techniques, such as 360° videos and 3D models. To the best of the authors’ knowledge, only two studies have compared 360° nature videos with a matching 3D model (Nukarinen et al. 2020; Yeo et al. 2020), and the findings in these studies indicate that the latter elicited (to some extent) more positive psychological responses than the former. Although these studies investigated such differences in a predominantly sedentary context (i.e., either while the participants sat on a chair (Nukarinen et al. 2020) or while they could stand and move in a limited space (Yeo et al. 2020), the findings of the present study are partly in line with this previous literature. More specifically, compared with control, the 3D model elicited higher levels of enjoyment, while the 360° video was associated with a slower walking speed. However, statistically significant differences were not observed for either of these measurements when comparing directly the 360° video and 3D model conditions. From the qualitative analysis, indications also emerged suggesting higher levels of presence and lower incidence of cybersickness in the 3D model compared to the 360° video, though these findings should be treated with caution, as they were not supported by the quantitative findings (albeit a non-significant statistical tendency was observed for the item “being there”). In particular, the qualitative reports indicated that some participants in the 360° video condition experienced cybersickness in the form of dizziness and challenges in maintaining balance while no participant in the 3D model condition mentioned such challenges. The potential for more issues related to cybersickness in the 360° video video would be in agreement with a recent review of factors influencing cybersickness, which highlighted that 360° videos are more susceptible to cybersickness than 3D models (Saredakis et al. 2020). Furthermore, researchers have proposed the idea that realistic looking simulations that fail to meet people’s expectations when it comes to accurate movement control may increase the risk of cybersickness (Venkatakrishnan et al. 2020). 360° videos are arguably realistic looking, which might make them susceptible to such effects. Although these issues related to cybersickness were minor in the present study it might have impacted other outcomes in the 360° video condition (namely enjoyment and walking speed), which emphasize the importance of identifying strategies to minimize negative aspects of VR exposure, such as cybersickness, in order to improve the effectiveness of experiences in VR.

### Strengths and limitations

Strengths of this study include the blinding of participants and examiner, and the controlled laboratory environment. The low levels of cybersickness should also be considered a strength, due its negative impact on other outcomes in similar studies. The only added benefit of virtual green exercise compared to control was higher levels of enjoyment following exposure to the 3D model. The lack of an experimental condition applying a virtual non-natural environment limits our ability to attribute this finding specifically to the environment and not to the VR-technology alone. This was in particular highlighted by the qualitative reports that the novelty of the technology contributed to the ratings of enjoyment. However, the lack of a similar improvement of enjoyment after the 360° video, and the qualitative reports that the natural elements in the virtual environments contributed to enjoyment, suggest that the novelty of VR in and of itself does not result in high levels of enjoyment. Another issue in the present study was that HR was not expressed as a percentage of each individual’s maximal heart rate, which limits its precision as a measure of exercise intensity. This was a calculated tradeoff because a measurement of maximal heart rate requires a highly intense and exhausting experience. In turn, this might have influenced the type of volunteers enlisting for this study and would definitely increase the load and the time commitment for the participants. Age-predicted maximal HR was not considered due to the limitations of these equations (Shookster et al. 2020).

## Conclusions

Both virtual green exercise conditions were generally well received by participants without causing concerning levels of negative side effects, such as cybersickness and negative affective responses. However, compared with treadmill walking with no exposure to VR, only one of the installations (the 3D model) provided some additional benefits (i.e., greater enjoyment), while the 360° video was associated with negative behavioral outcomes in the form of slower waking speed leading to reduced exercise output. Neither of the virtual conditions provided greater health benefits in terms of psychophysiological recovery compared with treadmill walking. Nevertheless, the findings of this study provide important insights regarding to the possibility of adopting virtual green exercise as a tool within health care settings, physical activity promotion, and research projects. In particular, the use of 3D model based virtual green exercise within physical activity promotion may be a promising area to explore, as enjoyment is an important component of exercise motivation. More research is, however, needed in this field, especially to establish possible long-term behavioral benefits of virtual green exercise interventions. This study also highlights limitations in the ability of virtual green exercise to fully reproduce the effects of experiences in real nature. Further research and optimization of virtual experiences of natural environments is required to achieve the full potential of this technology, preferably in accordance with current recommendations for this specific field of research (Joseph et al. 2020; Litleskare et al. 2020).

## Data Availability

The datasets generated during and analyze during the current study are available from the corresponding author on reasonable request.
